# Supplementary figures and images for: An emergency system for monitoring pulse oximetry, peak expiratory flow, and body temperature of patients with COVID-19 at home: Development and preliminary application
Source: PLoS One. 2021 Mar 26;16(3):e0247635. doi: 10.1371/journal.pone.0247635 (PMC7996990; doi:10.1371/journal.pone.0247635)

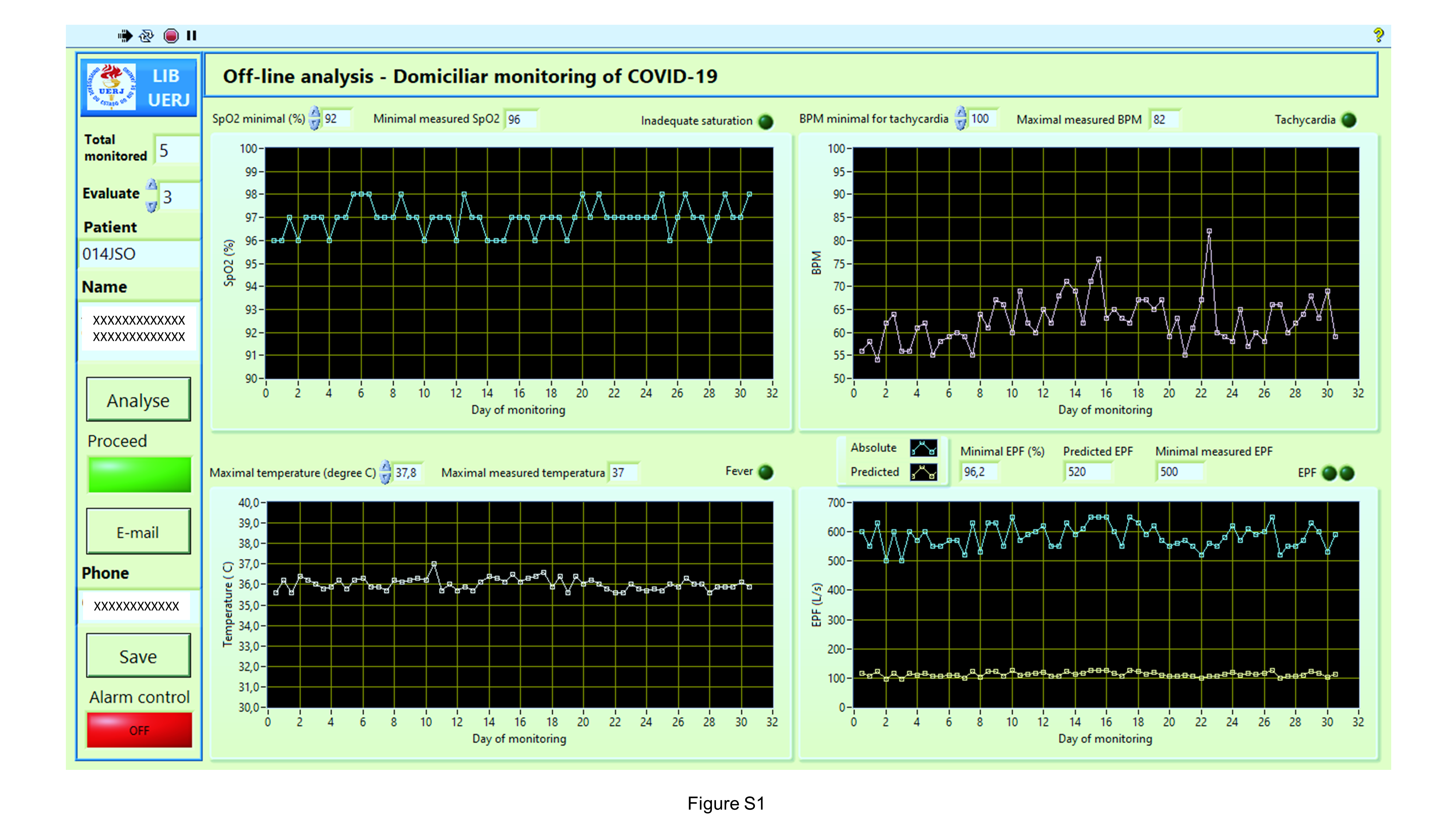

Supplement: S1 Fig — (TIF) [file pone.0247635.s001.tif]
