# Supplementary material for: An emergency system for monitoring pulse oximetry, peak expiratory flow, and body temperature of patients with COVID-19 at home: Development and preliminary application
Source: PLoS One. 2021 Mar 26;16(3):e0247635. doi: 10.1371/journal.pone.0247635 (PMC7996990; doi:10.1371/journal.pone.0247635)
Supplement: S1 Personal diary — (PDF) [file pone.0247635.s002.pdf]

HOSPITAL UNIVERSITÁRIO PEDRO ERNESTO

Diário de Monitoramento do Paciente

Paciente: ____Folha 1.4

Data do início do monitoramento: ____/_____/_____

	Oxigênio		Temp.	Pressão			Sopro
	SpO_2	BPM	Graus	Sistólica	Diastólica	Pulso	L/min
Dia 1 (06-10hs)							
Dia 1 (18-22hs)							
Dia 2 (06-10hs)							
Dia 2 (18-22hs)							
Dia 3 (06-10hs)							
Dia 3 (18-22hs)							
Dia 4 (06-10hs)							
Dia 4 (18-22hs)							
Dia 5 (06-10hs)							
Dia 5 (18-22hs)							
Dia 6 (06-10hs)							
Dia 6 (18-22hs)							
Dia 7 (06-10hs)							
Dia 7 (18-22hs)							

UNIVERSIDADE DO ESTADO DO RIO DE JANEIRO

Hospital Universitário Pedro Ernesto

Monitoramento dos pacientes – COVID-19

Paciente: ____ Folha 2.4

Data do início do monitoramento: ____/_____/_____

	Oxigênio		Temp.	Pressão			Sopro
	SpO_2	BPM	Graus	Sistólica	Diastólica	Pulso	L/min
Dia 8 (06-10hs)							
Dia 8 (18-22hs)							
Dia 9 (06-10hs)							
Dia 9 (18-22hs)							
Dia 10 (06-10hs)							
Dia 10 (18-22hs)							
Dia 11 (06-10hs)							
Dia 11 (18-22hs)							
Dia 12 (06-10hs)							
Dia 12 (18-22hs)							
Dia 13 (06-10hs)							
Dia 13 (18-22hs)							
Dia 14 (06-10hs)							
Dia 14 (18-22hs)							
Dia 15 (06-10hs)							
Dia 15 (18-22hs)							

UNIVERSIDADE DO ESTADO DO RIO DE JANEIRO
Hospital Universitário Pedro Ernesto
Monitoramento dos pacientes – COVID-19

Paciente: ____ Folha 3.4

Data do início do monitoramento: ____/_____/_____

	Oxigênio		Temp.	Pressão			Sopro
	SpO_2	BPM	Graus	Sistólica	Diastólica	Pulso	L/min
Dia 16 (06-10hs)							
Dia 16 (18-22hs)							
Dia 17 (06-10hs)							
Dia 17 (18-22hs)							
Dia 18 (06-10hs)							
Dia 18 (18-22hs)							
Dia 19 (06-10hs)							
Dia 19 (18-22hs)							
Dia 20 (06-10hs)							
Dia 20 (18-22hs)							
Dia 21 (06-10hs)							
Dia 21 (18-22hs)							
Dia 22 (06-10hs)							
Dia 22 (18-22hs)							
Dia 23 (06-10hs)							
Dia 23 (18-22hs)							

UNIVERSIDADE DO ESTADO DO RIO DE JANEIRO

Hospital Universitário Pedro Ernesto

Monitoramento dos pacientes – COVID-19

Paciente: ____ Folha 4.4

Data do início do monitoramento: ____/_____/_____

	Oxigênio		Temp.	Pressão			Sopro
	SpO_2	BPM	Graus	Sistólica	Diastólica	Pulso	L/min
Dia 24 (06-10hs)							
Dia 24 (18-22hs)							
Dia 25 (06-10hs)							
Dia 25 (18-22hs)							
Dia 26 (06-10hs)							
Dia 26 (18-22hs)							
Dia 27 (06-10hs)							
Dia 27 (18-22hs)							
Dia 28 (06-10hs)							
Dia 28 (18-22hs)							
Dia 29 (06-10hs)							
Dia 29 (18-22hs)							
Dia 30 (06-10hs)							
Dia 30 (18-22hs)							
Dia 31 (06-10hs)							
Dia 31 (18-22hs)							
